# Travel-associated chikungunya acquired in Myanmar in 2019

**DOI:** 10.2807/1560-7917.ES.2020.25.1.1900721

**Published:** 2020-01-09

**Authors:** Marta Díaz-Menéndez, Elena Trigo Esteban, Mugen Ujiie, Guido Calleri, Camilla Rothe, Denis Malvy, Emanuele Nicastri, Alfred L Bissinger, Marc Grandadam, Jonathan D Alpern, Federico Gobbi, Patricia Schlagenhauf, Alexandre Duvignaud, Emilie Javelle, Takato Nakamoto, Spinello Antinori, Davidson H Hamer

**Affiliations:** 1 National Referral Unit for Imported Tropical Diseases. Department of Internal Medicine. Hospital Universitario La Paz-Carlos III, IdiPAZ, Madrid, Spain; 2 These authors contributed equally to the work; 3 Vaccination Support Center, Disease Control and Prevention Center. National Center for Global Health and Medicine, Tokyo, Japan; 4 Travel Medicine Unit. Amedeo di Savoia Hospital. ASL Città di Torino, Torino, Italy; 5 LMU Hospital Centre, Division of Infectious Diseases and Tropical Medicine, Munich, Germany; 6 Department of Infectious Diseases and Tropical Medicine, CHU de Bordeaux & INSERM 1219, University of Bordeaux, Bordeaux, France; 7 National Institute for Infectious Diseases Lazzaro Spallanzani, IRCCS, Rome, Italy; 8 Institute for Tropical Medicine, Department of Internal Medicine, University Hospital Tübingen, Germany; 9 Arbovirus & Emerging viral diseases laboratory. Institut Pasteur du Laos, Vientiane, Laos; 10 Department of Travel and Tropical Medicine, HealthPartners; Department of Medicine, University of Minnesota, Minneapolis, United States; 11 Department of Infectious /Tropical Diseases and Microbiology, IRCCS Sacro Cuore Don Calabria Hospital, Negrar, Verona, Italy; 12 University of Zürich Centre for Travel Medicine, WHO Collaborating Centre for Travellers’ Health, Zürich, Switzerland; 13 Department of Infectious Diseases and Tropical Medicine, CHU de Bordeaux & INSERM 1219, University of Bordeaux, Bordeaux, France; 14 Laveran Military Teaching Hospital, Aix Marseille Univ, IRD, AP-HM, SSA, VITROME, Marseille, France; 15 Disease Control and Prevention Center, National Center for Global health and Medicine, Tokyo, Japan; 16 Department of Biomedical and Clinical Sciences “L Sacco”, University of Milan. Tropical Medicine Unit, ASST Fatebenefratelli Sacco, Milan, Italy; 17 Department of Global Health and Boston University School of Public Health and Section of Infectious Diseases, Department of Medicine, Boston University School of Medicine, Boston, MA, United States

**Keywords:** Chikungunya virus, Outbreak, travel medicine, surveillance

## Abstract

Eighteen cases of chikungunya virus infection in travellers returning from Myanmar were reported to the GeoSentinel Surveillance Network, its subnetwork EuroTravNet and TropNet in 2019, reflecting an ongoing local outbreak. This report reinforces the importance of travellers as sentinels of emerging arboviral outbreaks and highlights the importance of vigilance for imported cases, due to the potential for dissemination of the virus into areas with competent local vectors and conducive environmental conditions.

In early October 2019, a GeoSentinel Surveillance Network site in Madrid, Spain, identified two patients with chikungunya virus (CHIKV) infection who had recently visited Myanmar. Rapid outreach to GeoSentinel sites (including EuroTravNet), TropNet and Laos external collaborators identified 16 additional infected travellers who acquired CHIKV infection in Myanmar during 2019.

According to a newspaper report, the Myanmar Department of Public Health identified an outbreak of CHIKV infection in 2019, mainly in Nay Pyi Taw, Kachin State, and Tanintharyi Region, Myanmar [1]. In the previous 8 years however, no cases of CHIKV infection had been officially reported in the country [2,3].

We describe travellers with imported CHIKV infection from Myanmar who were diagnosed in 2019 (January to November) and identified at GeoSentinel, EuroTravNet and TropNet sites. Some epidemiological and clinical information as well as places visited by the travellers are presented.

## Case finding

GeoSentinel is a global surveillance network for emerging infectious diseases that has 68 sites in 28 countries; EuroTravNet is its European subnetwork; TropNet represents a separate European surveillance entity represented by 75 specialised tropical medicine centres in Europe.

For chikungunya surveillance, GeoSentinel follows case definitions proposed by the World Health Organization (WHO) Regional Office for Southeast Asia [4]. Such definitions were used in the current report. A possible case was a patient with acute onset of fever >38.5°C and severe arthralgia/arthritis not explained by other medical conditions. A probable case was a patient meeting both the previously mentioned clinical criteria and the following epidemiological criteria: residing or having visited epidemic areas, having reported transmission within 15 days prior to the onset of symptoms. A confirmed case had to meet one or more of the following laboratory criteria, irrespective of the clinical presentation: (i) virus isolation in cell culture or animal inoculations from acute phase sera, or (ii) presence of viral RNA in acute phase sera as demonstrated by RT-PCR, or (iii) presence of virus-specific IgM antibodies in single serum sample in acute or convalescent stage, or (iv) fourfold increase in virus-specific IgG antibody titre in samples collected at least 3 weeks apart.

For this study, cases were excluded if they were not in Myanmar at the likely time of exposure, which was inferred from their date of symptom onset and the typical incubation period (defined as 3 to 7 days) [5] or if the travellers had more than one potential travel destination exposure based on the incubation period. 

## Description of cases

Epidemiological and clinical details for each patient were collected from reporting sites. 

The 18 cases reported in the current study had a median age of 51 years (range: 19–68 years) and 10 were male (Table 1). Among the overall cases, one had visited friends and relatives in Myanmar at the likely time of exposure, two were there on a business trip and three were expatriates. The rest of the cases, which represented the majority (n = 12), were tourists who had experienced a median length of stay of 13 days in Myanmar (range: 6–65 days).

**Table 1:** Epidemiological and travel characteristics of confirmed and probable chikungunya cases among travellers returning from Myanmar, 2019 (n = 18)

Case	Reporting country	Places visited in Myanmar	Pre-travel consultation	Period of stay in Myanmar (length of exposure period)	Approximate age in years^a^	Underlying medical condition(s)	Month of symptom onset
**1**	Japan	Yangon	Yes	Apr 2017 to Jul 2019 (NA)	60	Hypertension	Jul 2019
**2**	Japan	Yangon, Naypyidaw	No	Aug 2019 (6 days)	25	Atopic dermatitis	Aug 2019
**3**	France	Shan state, Bagan, Yangon	Yes	Jul 2019 (12 days)	45	No	Aug 2019
**4**	Italy	Nyaungshwe	Yes	Lived in Myamnar until Aug 2019 (NA)	60	No	Aug 2019
**5**	Japan	Yangon	Yes	Aug 2018 to Aug 2019 (NA)	70	Hyperlipidaemia, hypertension, diabetes mellitus	Aug 2019
**6**	France	Yangon, Hpa-An, Moulmein, Ye, Mandalay, Bagan, Inle lake, Pindaya	Yes	Jul to Aug 2019 (23 days)	45	No	Aug 2019
**7**	United States	Ye, (Mon State), Yangon	Unknown	May to Aug 2019 (65 days)	20	No	Jul 2019
**8**	Spain	Yangon, Mandalay, Inle Lake	No	Aug 2019 (7 days)	30	No	Aug 2019
**9**	Spain	Yangon Mandalay, Inle Lake, Bagan	No	Aug 2019 (14 days)	55	No	Aug 2019
**10**	Italy	Yangon, Mandalay, Inle Lake,	No	Aug to Sep 2019 (12 days)	50	No	Sep 2019
**11**	Italy	Yangon, Mandalay, Inle Lake	No	Aug to Sep 2019 (12 days)	50	No	Sep 2019
**12**	Laos	Mandalay	No	Sep 2019 (12 days)	50	No	Sep 2019
**13**	Germany	Yangon, Inle Lake, Bagan, Mandalay	Yes	Jan 2019 (15 days)	65	Mitral valve disease	Feb 2019
**14**	Germany	Yangon, Inle Lake, Bagan, Mandalay	Yes	Jan 2019 (15 days)	30	No	Feb 2019
**15**	Italy	Yangon, Bagan, Mandalay	No	Jul to Aug 2019 (12 days)	65	Hypertension	Jul 2019
**16**	Italy	Rangoon, Bagan	No	Aug 2019 (10 days)	55	No	Aug 2019
**17**	Germany	Yangon, Inle Lake, Mandalay, Bagan, Thandwe/West Coast	No	Oct 2019 (16 days)	25	No	Oct 2019
**18**	France	Yangon, Mandalay, Bagan, Inle Lake	Unknown	Oct to Nov 2019 (15 days)	55	No	Oct 2019

F: female; Ig: immunoglobulin; M: male; NA: not assessed; VFR: visiting friends and relatives.

^a^ Age rounded by five.

The majority of cases were imported to Europe (n = 13) and Asia (n = 4), with one case in the Americas. Fewer than half (7/16) of travellers with available information, received pre-travel advice. Destinations visited in Myanmar are shown in the Figure.

**Figure:**
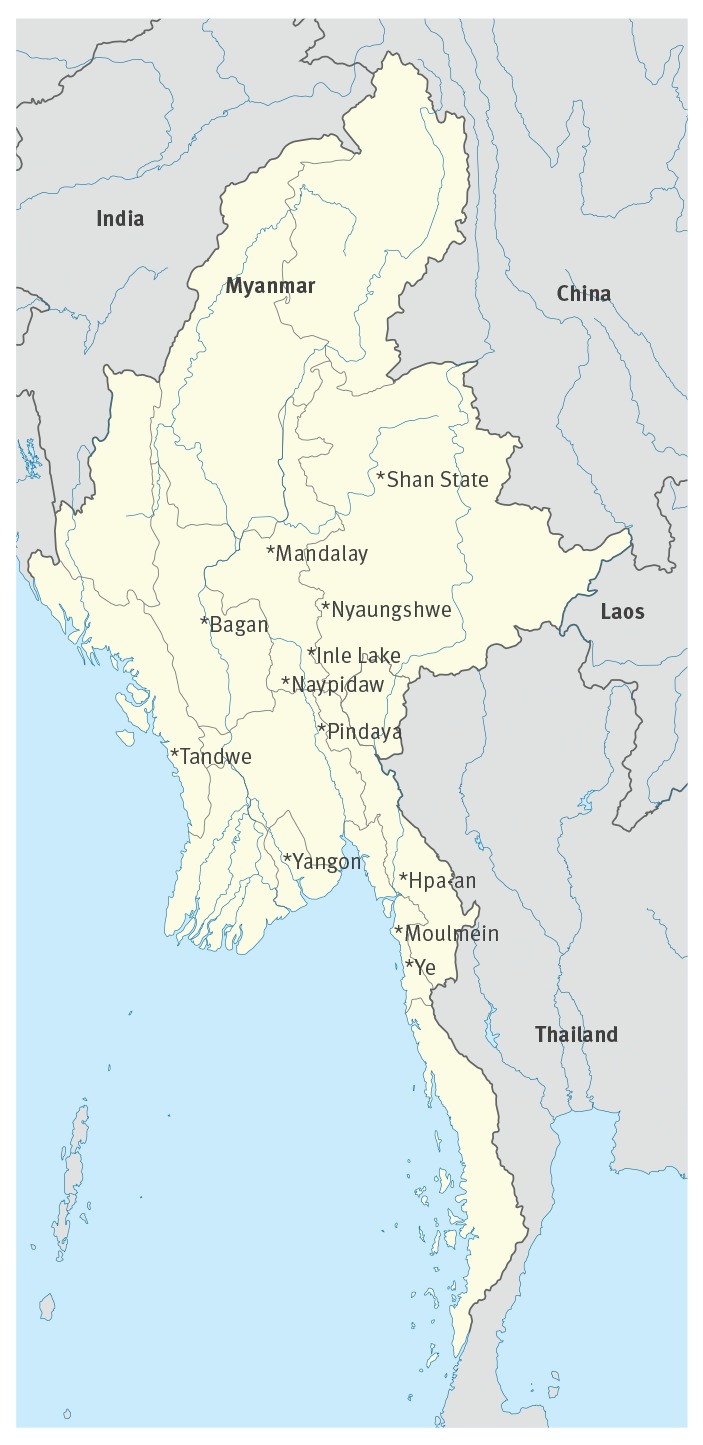
Possible places of exposure: destinations visited by travellers before chikungunya virus infection, Myanmar, 2019 (n = 18)

Asterisks represent the places where travellers in the current study were possibly exposed to chikungunya virus. Some travellers visited the same destinations.

Source: Map adapted from Wikimedia Commons, the free media repository: https://commons.wikimedia.org/wiki/File:Myanmar_location_map.svg

Among the 18 cases, acute symptoms reported included fever (n = 18), arthralgia (n = 16) and rash (n = 14) (Table 2). A median of 9.5 days (range: 1–240 days) elapsed between symptom onset and diagnosis. Most travellers (n = 13) were identified at reporting sites between August and October 2019. Seven cases were confirmed by PCR, including five who were also found IgM positive, and nine cases were confirmed by a positive IgM only; the remaining two patients had probable diagnoses based on a single IgG positive serology. No acute coinfections with other arboviruses were described in the 16 patients tested for additional pathogens.

**Table 2:** Clinical and diagnostic characteristics of confirmed and probable chikungunya cases among returning travellers from Myanmar, 2019 (n = 18)

Case	Clinical acute symptoms	Outcome	Need of hospitalisation (country)	Clinical status as at 20 Oct 2019	Chikungunya diagnostic method	Day of diagnosis in 2019 (time from onset of illness)	Other arboviral diagnostic test results
**1**	Fever, rash, arthralgias (fingers, wrists, ankles, knees)	Persistent arthralgias needing NSAIDs	No	Unknown	IgM+ (N Health Myanmar Co., Ltd, Yangon, Myanmar)	08 Jul (6 days)	Dengue: IgM−/IgG−; NS1− Zika: NS1−
**2**	Fever, rash, arthralgia (wrists, ankles), retro-orbital pain, conjunctival hyperaemia	Persistent arthralgias needing NSAIDs	No	Improvement of arthralgias	IgM+; PCR+ (NovaLisa, NovaTec, Dietzenbach, Germany)	13 Aug (6 days)	Dengue: PCR−; IgM− Zika: PCR−; IgM−
**3**	Fever, rash, arthralgia, myalgia, conjunctivitis	Persistent incapacitating arthralgias needing NSAIDs	No	Mild and improving arthralgia, no more NSAIDs needed	RT-PCR+ (RealStar chikungunya RT-PCR, Altona diagnostics, Hamburg, Germany)	14 Aug (1 day)	Dengue: PCR− Zika: PCR−
**4**	Fever, arthralgia, deep asthenia, profuse sweating	Persistent arthralgias needing NSAIDs and CT (prednisone)	Yes (Italy)	Unknown	IgM+/IgG+ (Euroimmun, Luebeck, Germany)	14 Aug (9 days)	Unknown
**5**	Fever, rash, arthralgia (fingers, wrists, knees), ankle swelling, conjunctival hyperaemia	Persistent arthralgias needing NSAIDs	Yes (United States)	Improving existing arthritis and arthralgia	IgM+/IgG+ (NovaLisa, NovaTec )	21 Aug (20 days)	Dengue: IgM−/IgG− Zika: IgM−
**6**	Fever, ankles oedema	Persistent and recurrent arthralgias needing NSAIDs; fatigue, headache	No	Persisting moderate arthralgia, still needing NSAIDs	IgM+/IgG+ (Euroimmun)	03 Sep (12 days)	Dengue: IgM− Zika: IgM−
**7**	Fever, rash, chills, myalgias, symmetric arthralgias (knees, ankles, wrists, hands)	Improving with NSAIDs	No	Improving	IgM+/IgG+ (Arup Laboratories, Utah, United States)	04 Sep (41 days)	Not tested
**8**	Fever, rash, arthralgias (ankles, wrists, feet, interphalangeal joints in hands, bilateral)	Persistent arthralgias needing NSAIDs and CT	No	Improved	IgM+ (Euroimmun)	09 Sep (10 days)	Dengue: IgM−/IgG− Zika: IgM ambiguous/IgG−
**9**	Fever, rash, arthralgia	Persistent arthralgias needing NSAIDs and CT	No	Improved	IgM+ (Chemiluminescence Virclia UNILABS, Madrid, Spain)	13 Sep (21 days)	Dengue: IgM−/ IgG− Zika: IgM−/ IgG−
**10**	Fever, rash, arthralgia, diarrhoea, paraesthesia lower limbs, swelling upper and lower limbs, lymphadenopathy	Persistent arthralgias needing NSAIDs	No	Persistent arthralgia	IgM+/IgG−; PCR+ (ChLIA Alifax, Polverada, Italy)	13 Sep (7 days)	Dengue: RDT− Zika: PCR−; IgM−/ IgG−
**11**	Fever, rash, arthralgia, diarrhoea	Persistent arthralgias needing NSAID	No	Persistent arthralgia	IgM+/IgG-; PCR+ (ChLIA Alifax)	13 Sep (6 days)	Dengue: RDT− Zika: PCR−; IgM−/IgG−
**12**	Fever, pruritus, rash, head and limbs felt swollen	Persistent arthralgias needing paracetamol	No	Little light headed when exercise	RT-PCR+ (in house RT-PCR: [24])	23 Sep (4 days)	Dengue NS1−; RT−PCR− Zika: not tested
**13**	Fever, nausea, arthralgias, ankle oedema	Persistent incapacitating arthralgias and ankle oedema needing NSAIDs and CT	No	Persistent incapacitating arthralgias and ankle oedema needing NSAIDs and corticosteroids	IgG+ (Euroimmun)	07 Oct (8 months)	Dengue: NS1−; IgM−, IgG+ (multiple exposures before, IIFT+ with low titre) Zika: not tested
**14**	Fever, rash, arthralgia	Not needing any treatment	No	Mild ankles arthralgias	IgG+ (Euroimmun)	07 Oct (8 months)	Dengue: NS1−; IgM−/IgG– Zika: not tested
**15**	Fever, rash, arthralgias (wrists, right elbow, metacarpophalangeal joints), general malaise and nausea	Persistent arthralgias needing NSAIDs	Yes (Myanmar)	Improve	IgM+/IgG+ (NovaLisa, NovaTec)	14 Oct (74 days)	Dengue: NS1− Zika: not tested
**16**	Fever, rash, arthralgia	Persistent arthralgias needing NSAIDs	No	Persistent arthralgias	IgM+/IgG+ (Euroimmun)	16 Oct (60 days)	Not tested
**17**	Fever, rash, arthralgias (hands, ankles)	No drugs needed	No but presented to local outpatient clinic where no testing for CHIK was done.	Mild arthralgias in fingers and ankles	RT-PCR+; IgM+/IgG– (Fast Track Diagnostics. Ltd, Esch-sur-Alzette, Luxembourg)	21 Oct (7 days)	Zika: PCR–; IgM–/IgG– Dengue: PCR−; NS1–
**18**	Fever, arthralgia	Improving arthralgia, NSAIDs no more needed	Yes (France)	Moderate persisting arthralgias	RT-PCR+ (RealStar chikungunya RT-PCR, Altona diagnostics); IgM+/IgG– (Euroimmun)	02 Nov (4 days)	Dengue: PCR–; IgM– Zika: PCR–; IgM–

CT: corticosteroids; IIFT: indirect immunofluorescence test; Ig: immunoglobulin; NSAID: non-steroidal anti-inflammatory drug; NS1: nonstructural 1 protein antigen; RDT: rapid diagnostic test; RT-PCR: real time PCR.

Four patients were hospitalised, and 15 needed non-steroidal anti-inflammatory drugs; four had corticosteroids added to manage symptoms (Table 2). As at 20 October 2019, fourteen patients still had persistent symptoms, predominantly arthralgia.

## Discussion

Chikungunya is often a mild self-limited illness, but severe and life-threatening complications have been described [6]. Persistent polyarthralgia can affect up to 40% of infected individuals and may last for months or years [7,8]. A high proportion of cases in our case series (14 of 18) had persistent sequelae (mainly arthralgia) that interfered with their daily life. Given the potential for long-term morbidity, the absence of a curative treatment or a preventive vaccine, detailed pre-travel counselling should focus on mosquito-bite prevention, particularly for those at high risk of incapacitating complications such as women older than 40 years, people with underlying rheumatic diseases, and professional athletes [9].

Considering the short duration of stay in different parts of Myanmar of many of the currently reported travellers, as well as the usual incubation period of chikungunya, the specific place where they were infected cannot be determined with certainty. Most patients in the case series acquired chikungunya from August to October, likely due to enhanced vector activity at the end or after the monsoon season, which occurs between May and August.

CHIKV has been detected in South and South East Asia since the 1950s [3], but its current epidemiology is poorly understood [10]. In Myanmar, CHIKV infection was first described in 1973 [11]. Since this time, the virus has caused outbreaks in 1998, 2006 and 2008 [3]. The number of reported cases subsequently declined until 2010, when local surveillance identified the last reported case [3]. Thereafter, from 2011 to 2018 no CHIKV cases were officially reported in the country, however a review of travellers who acquired chikungunya in Myanmar in the GeoSentinel database from 2000 to October 2019 revealed two cases in 2015 and 2016, one probable and one confirmed.

In 2019, Myanmar’s National Health Laboratory detected chikungunya cases again [1]. During the same year, other national surveillance systems also identified travellers who acquired chikungunya in Myanmar. For example, the Japanese National Institute of Infectious Diseases, which systematically tracks travellers, detected 28 imported cases of chikungunya from Myanmar to Japan [12]. The Italian Arboviriasis Surveillance System also detected four imported cases in Italy [13]. Our report highlights additional cases of exported CHIKV infection from Myanmar.

Little is known about factors contributing to the multiple detections of CHIKV infections in or from Myanmar in 2019. While aside from this year, no cases within the country had been officially observed since the end of 2010, the detection of exported cases in 2015 and 2016 could suggest that CHIKV might have continued to circulate in Myanmar after 2010. Myanmar’s disease surveillance system includes a Central Epidemiology Unit (CEU) and several vertical control programmes [14]. Certain areas of the country are, however, difficult to access and there could have been underreporting due to limitations of surveillance capacity.

Myanmar has geographical and epidemiological characteristics that put it at risk for CHIKV epidemics: a highly susceptible population [15], a long border with two large neighbouring endemic countries (India and Thailand) with cross-border population movement for trade and travel purposes [16], and competent vectors (both *Aedes aegypti* and *Ae. albopictus)* [17]. According to the Thai Ministry of Public Health, CHIKV is currently circulating in Thailand with an increasing incidence: as at 30 October 2019, the country has reported 8,744 cases this year [18]. Coincidentally, GeoSentinel recently documented a rise in the number of travel-associated CHIKV infections from Thailand [19]. Without further epidemiological and phylogenetic evidence, it remains difficult to establish whether any links exist between cases in Thailand and those in Myanmar.

It should be noted that Myanmar is an increasingly popular tourist destination, with more than 3.5 million visitors in 2017 [20]. Hence, although we report more cases exported from Myanmar in 2019 than in previous years, a surge in tourism to this country may have led to an apparent increase of the number of exported cases. GeoSentinel data are moreover not population-based so rates or risk estimates cannot be derived. Also, diagnostics depend on local site interpretation and reporting.

Imported CHIKV infection by viraemic travellers returning to their home countries raises the possibility of virus spread to these countries if competent vectors are present, mainly in hot seasons, as European *Ae. albopictus* is affected by seasonal temperature and undergoes a winter diapause [21]. There is also a potential risk of transmission by other routes, such as blood donation [22]. In Europe, some countries (Italy in 2007 and 2017, France in 2010, 2014 and 2017) have experienced autochthonous transmission of CHIKV through viraemic travellers [23]. Surveillance and early detection of both imported and autochthonous CHIKV infections is therefore relevant in areas with competent vectors, as well as close vector monitoring and rapid public health response.

This report of imported CHIKV infections reinforces the importance of travellers as sentinels of local outbreaks, particularly in settings with limited public health surveillance and reporting infrastructure. 
